# Ist der Einsatz digitaler Technologien der Gamechanger für die chirurgische Weiterbildung der Zukunft? Eine deutschlandweite Analyse

**DOI:** 10.1007/s00104-025-02306-y

**Published:** 2025-05-22

**Authors:** Dolores T. Krauss, Hans F. Fuchs, Sebastian Schaaf, Sabine Drossard, Romina Rösch, Beate Blank, Christiane J. Bruns, Udo Rolle, Thomas Schmitz-Rixen, Juliane Kröplin

**Affiliations:** 1 Klinik und Poliklinik für Allgemein‑, Viszeral‑, Thorax- und Transplantationschirurgie, Medizinische Fakultät und Uniklinik Köln, Universität zu Köln, Kerpener Str. 62, 50937 Köln, Deutschland; 2 Viszeral- und Thoraxchirurgie, Bundeswehrzentralkrankenhaus Koblenz, Perspektivforum Junge Chirurgie der Deutschen Gesellschaft für Chirurgie und Klinik für Allgemein-, Koblenz, Deutschland; 3 Klinik und Poliklinik für Allgemein‑, Viszeral‑, Transplantations‑, Gefäß- und Kinderchirurgie, Universitätsklinikum Würzburg, Oberdürrbacher Str. 6, 97080 Würzburg, Deutschland; 4 Thoraxklinik Heidelberg der Universitätsklinik Heidelberg, Röntgenstraße 1, 69126 Heidelberg, Deutschland; 5 Dr. Erler Kliniken, Handchirurgie, Plastische und Mikrochirurgie, Kontumazgarten 4–19, 90429 Nürnberg, Deutschland; 6 Klinik für Kinderchirurgie und Kinderurologie, Universitätsklinikum Frankfurt, Theodor-Stern-Kai 7, 60590 Frankfurt am Main, Deutschland; 7 Deutsche Gesellschaft für Chirurgie e. V., Berlin, Deutschland; 8 Klinik für Mund‑, Kiefer- und Plastische Gesichtschirurgie, Universitätsmedizin Rostock, Schillingallee 35, 18057 Rostock, Deutschland

**Keywords:** Medizintechnik, Robotik, Digitalisierung, Künstliche Intelligenz, Ausbildung, Medical technology, Robotics, Digitalization, Artificial intelligence, Surgical training

## Abstract

**Hintergrund:**

Der Einsatz digitaler Technologien gewinnt in der Medizin zunehmend an Bedeutung und beeinflusst maßgeblich die Entwicklungen in der Chirurgie. In der chirurgischen Aus- und Weiterbildung besteht jedoch ein großer Nachholbedarf, um junge Chirurg:innen adäquat auf die damit verbundenen Herausforderungen vorzubereiten.

**Ziel der Arbeit:**

Ziel der vorliegenden Studie ist die Analyse von Bedeutung, Einsatz und Einfluss des Einsatzes digitaler Technologien auf die Attraktivität als Weiterbildungsstandort in der Chirurgie in Deutschland.

**Material und Methoden:**

Von April bis September 2024 erfolgte die Durchführung einer Onlinebefragung mit insgesamt 12 offenen (*n* = 2) und standardisierten (*n* = 10) Fragen. Die geschlossenen Fragen konnten auf einer Likert-Skala von ein 1 (trifft vollkommen zu) bis 5 (trifft überhaupt nicht zu) beantwortet werden. Der Fragebogen wurde über den E‑Mail-Verteiler der Deutschen Gesellschaft für Chirurgie (DGCH) und deren Social-Media-Kanäle versandt.

**Ergebnisse:**

Insgesamt 97 Antwortdatensätze wurden analysiert. Die Mehrzahl der Teilnehmenden war zum Analysezeitpunkt in der Viszeralchirurgie tätig, (*n* = 54, 64 %) überwiegend an nichtuniversitären Kliniken (*n* = 49, 58 %). 19 % befanden sich in Weiterbildung. Bei der Wahl der aktuellen Tätigkeitsstätte legten 44 % viel Wert auf eine fortgeschrittene Digitalisierung. 61 % gaben an, generative KI noch gar nicht genutzt zu haben. Zugang zu einer kurrikulären Weiterbildung an OP-Robotern haben nur 9 % der Ärzt:innen in Weiterbildung (ÄiW). Ein Standortwechsel zu einem Standort mit mehr Medizintechnik kommt für 19 % in Betracht. Während 26 % der Studienteilnehmer:innen sich selbst von einem KI-basierten Roboter operieren lassen würden, können sich 46 % der Teilnehmenden vorstellen, diese Technik bei ihren Patient:innen zu nutzen.

**Diskussion:**

Die vorliegende Analyse gibt einen Einblick über die Bedeutung und den Einsatz digitaler Technologien in der Chirurgie in Deutschland. Es zeigen sich insbesondere Defizite bei der Anwendung KI-basierter Methoden, einer flächendeckenden Versorgung mit digitalen Technologien sowie dem Zugang von ÄiW zu einer innovativen kurrikulären Weiterbildung. Die Ergebnisse bestätigen zudem die Notwendigkeit, das Bewusstsein für die Thematik weiter zu steigern und die Reichweite der DGCH über die sozialen Medien zu erhöhen.

**Graphic abstract:**

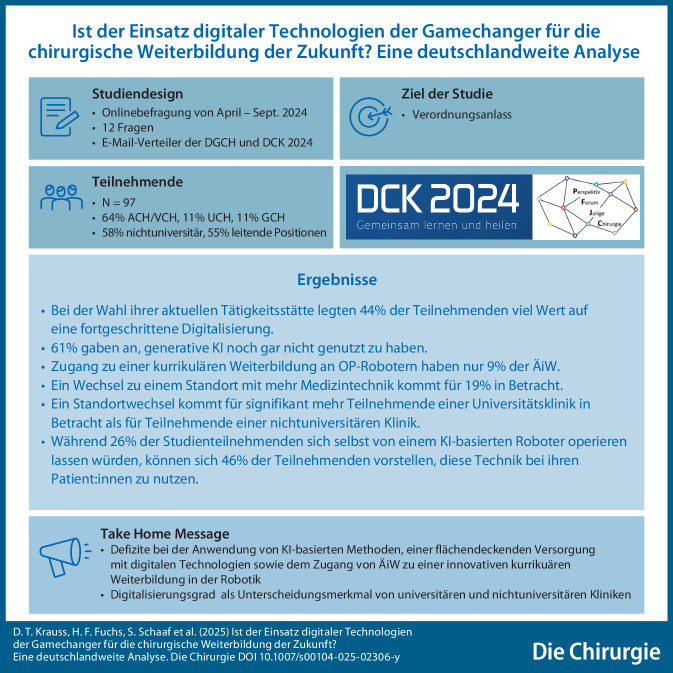

## Abstract (en)

**Background:**

The use of digital technologies is becoming increasingly important in medicine and is having a significant impact on developments in the surgical field. However, there is a great need to improve and implement those new techniques in surgical education and training in order to adequately prepare young surgeons for associated challenges.

**Objectives:**

The aim of this study is to analyze the importance, use, and influence of digital technology on the success of future surgical training in Germany.

**Materials and methods:**

An online survey was conducted from April–September 2024 with a total of 12 open (*n* = 2) and standardized (*n* = 10) questions. The closed questions could be answered on a Likert scale from 1 (strongly agree) to 5 (strongly disagree). The questionnaire was sent out via the email distribution list of the German Society of Surgery and its social media channels.

**Results:**

A total of 97 response datasets were analyzed. At the time of analysis, the majority of participants were working in general surgery (*n* = 54, 64%) and at a nonuniversity clinic (*n* = 49, 58%). In all, 19% of the respondents were residents. When choosing their current workplace, 44% prioritized advanced digitalization, while 61% stated that they had not yet used generative AI at all. Only 9% of trainees had access to curricular training on robotic systems. A change to a location with more advanced medical technology was considered by 19%. While 26% of study participants would consider being operated on by an AI-assisted robotic system, 46% of the participants could imagine using this technology on their patients.

**Conclusion:**

This analysis provides insight into the importance and use of digital technology in surgery in Germany. In particular, it reveals deficits in the use of AI-based methods, comprehensive provision of digital technologies, and the access of trainees to innovative training. The results also confirm the need to further raise awareness of the topic.

Die digitale Transformation erfasst zunehmend die Medizin und ist insbesondere für die Chirurgie, die sich in enger Anlehnung an den Fortschritt digitaler Technologien weiterentwickelt, von großer Bedeutung. Die Vorteile, aber auch Risiken, die sich durch den Einsatz digitaler Technologien ergeben, sind vielfach beschrieben und erfordern einen reflektierten und verantwortungsbewussten Einsatz. Die Integration dieser in den chirurgischen Alltag stellt Chirurg:innen vor neue Herausforderungen, denen insbesondere durch umfassende digitale Kompetenzen der Anwender begegnet werden muss. Der chirurgischen Aus- und Weiterbildung kommt hierbei eine besondere Bedeutung zu.

## Hintergrund und Fragestellung

Die Einbindung robotergestützter Systeme in der Chirurgie ist in den letzten Jahren bereits Teil der Routinepatientenversorgung geworden [1]. Zudem revolutionieren die Weiterentwicklung künstlicher Intelligenz (KI), virtueller Realität (VR) und augmentierter Realität (AR) die Wissenschaft. Seit der Einführung von ChatGPT im November 2022 hat sich der Einsatz generativer KI rasant weiterentwickelt. Eine Vielzahl möglicher Anwendungsbereiche betrifft neben Wissenschaft, Forschung und Lehre auch die Patientenversorgung [2, 3]. Insbesondere in der Aus- und Weiterbildung haben Large Language Models (LLM) wie ChatGPT ein großes Potenzial, da sie Ärzt:innen bei der Diagnosestellung und klinischen Entscheidungsfindung unterstützen und so Lernerfolge und die Patientenversorgung verbessern können [4]. Zudem ergeben sich breite Anwendungsgebiete, die bei administrativen Prozessen unterstützen und Freiräume für die Behandlung von Patient:innen schaffen können.

Trotz der zunehmenden Verfügbarkeit und des Einsatzes digitaler Technologien in der Chirurgie gibt es nach wie vor erhebliche Defizite in der chirurgischen Aus- und Weiterbildung, was den gezielten und strukturierten Erwerb digitaler Kompetenzen betrifft [5]. Auch wenn sowohl Ärzt:innen in Weiterbildung (ÄiW) als auch Weiterbildende den Beginn der robotischen Ausbildung bereits zu Beginn der Weiterbildung präferieren, ist die Realität eine andere [6, 7]. Aktuell existieren keine Daten zur Verbreitung von Konsolentraining in der chirurgischen Weiterbildung in Deutschland. Angesichts dieser Entwicklungen wird es zunehmend wichtiger, den Einfluss digitaler Technologien auf die chirurgische Praxis zu untersuchen und deren Rolle in der chirurgischen Weiterbildung zu bewerten. Die vorliegende Studie zielt darauf ab, den Einsatz digitaler Technologien in der chirurgischen Weiterbildung in Deutschland aus Sicht klinisch tätiger Chirurg:innen zu erfassen und zu analysieren, wie diese Technologien die Attraktivität von Weiterbildungsstandorten und eine potenzielle Personalfluktuation beeinflussen.

## Studiendesign und Untersuchungsmethoden

Von April bis September 2024 erfolgte die Durchführung einer Onlinebefragung im Rahmen des Deutschen Chirurgie Kongresses 2024. Es wurde ein onlinebasierter elektronischer Fragebogen mit der Umfrageplattform LimeSurvey (LimeSurvey GmbH, Hamburg, Deutschland) erstellt, welcher insgesamt 12 Fragen enthielt, hiervon 2 offene und 10 standardisierte Fragen (Tab. 1; [8]). Die geschlossenen Fragen konnten auf einer Likert-Skala von ein 1 (trifft vollkommen zu) bis 5 (trifft überhaupt nicht zu) beantwortet werden. Der Fragebogen wurde über den E‑Mail-Verteiler der Deutschen Gesellschaft für Chirurgie und deren Social-Media-Kanäle versandt. Die Teilnahme an der Umfrage erfolgte anonym und freiwillig. Es wurden keine personenbezogenen Daten gespeichert. Die Umfrage enthielt drei demographische Fragen zur Zuordnung der Fachdisziplin, dem Tätigkeitsbereich sowie der Karrierestufe. Zwei Fragen beschäftigten sich mit dem Stand und der Bewertung der Wichtigkeit der Digitalisierung. Zwei weitere Fragen erfassten den Einsatz von OP-Robotern in der Aus- und Weiterbildung und die Wichtigkeit digitaler Technologien für den Standort. Die offenen Fragen gaben den Teilnehmenden die Möglichkeit, zu eben diesen Themenkomplexen aktuelle und gewünschte Anwendungsbereiche anzugeben. Eine Frage zielte auf die Erfassung des Einsatzes von generativer KI und ChatGPT ab und die letzten zwei Fragen erfassten, ob Teilnehmende sich selbst durch KI-basierte Robotik operieren lassen oder diese einsetzen würden.

**Tab. 1:** Die 12 Fragen der Onlineumfrage

1. Chirurgische Fachdisziplin (Single Choice)
2. Tätigkeitsbereich (Single Choice)
3. Karrierestufe (Single Choice)
4. Bei der Wahl meiner aktuellen Tätigkeitsstätte habe ich viel Wert auf eine fortgeschrittene Digitalisierung (z. B. digitale Patientenakte, OP-Roboter) meiner Abteilung gelegt. (Likert-Skala 1–5)
5. Im Rahmen meiner ärztlichen Tätigkeiten/Studium habe ich generative KI (z. B ChatGPT) bereits genutzt im Rahmen von: (Multiple Choice)
6. Die Planung, Durchführung und Evaluation operativer Eingriffe erfolgt in meiner Abteilung digital. (Likert-Skala 1–5)
7. Im Verlauf der Weiterbildung erfolgt eine kurrikuläre Einarbeitung an OP-Robotern. (Single Choice)
8. Ich habe bereits darüber nachgedacht, meine Tätigkeitsstätte zu wechseln, an einen Standort an dem mehr Medizintechnik zum Einsatz kommt. (Likert-Skala 1–5)
9. In welchen Weiterbildungsbereichen werden digitale Lösungen/Medizintechnik in Ihrer Abteilung bereits genutzt? (Freitext)
10. In welchen Weiterbildungsbereichen würden Sie sich eine Entlastung durch digitale Lösungen/Medizintechnik wünschen? (Freitext)
11. Würden Sie sich selbst durch KI-basierte Robotik operieren lassen (analog zum selbstfahrenden Auto)? (Single Choice)
12. Würden Sie selbst mithilfe KI-basierter Robotik operieren wollen? (Single Choice)

Die statistische Auswertung erfolgte mittels Excel (Microsoft, Redmond, WA, USA) sowie SPSS (IBM SPSS Statistics, Armonk, New York, USA).

## Ergebnisse

**Abb. 1:**
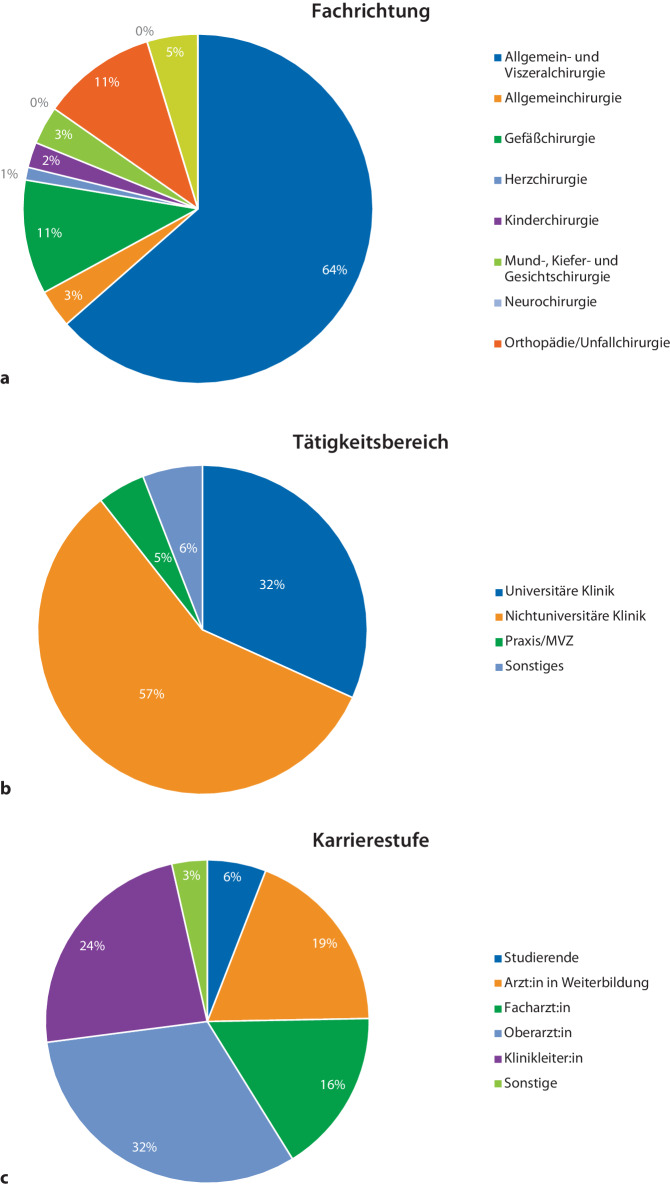
**a** Fachrichtung. **b** Tätigkeitsbereich. **c** Karrierestufe der Studienteilnehmer (in %)

Es nahmen insgesamt 97 Chirurg:innen an der Umfrage teil. 12 Teilnehmende beendeten die Umfrage nicht, sodass insgesamt 85 vollständige Antwortdatensätze analysiert werden konnten. Die Mehrzahl der Teilnehmenden war zum Analysezeitpunkt in der Viszeralchirurgie tätig (*n* = 54, 64 %). Die zweithäufigsten Fachdisziplinen waren die Orthopädie/Unfallchirurgie sowie die Gefäßchirurgie (je *n* = 9, 11 %; Abb. 1a). Insgesamt waren 49 (58 %) der Teilnehmenden an einer nichtuniversitären Klinik tätig, 27 (32 %) an einer universitären Klinik (Abb. 1b); 16 Teilnehmende (19 %) befanden sich in Weiterbildung, 47 Teilnehmende (55 %) hatten leitende Positionen inne (Abb. 1c).

Insgesamt 21 % (*n* = 18) der Teilnehmenden legten bei ihrer Wahl der Tätigkeitsstätte viel Wert auf eine fortgeschrittene Digitalisierung, 19 % (*n* = 16) legten hierauf keinen Wert. Insgesamt legten 44 % (*n* = 37) Wert auf eine Digitalisierung im Vergleich zu 46 % (*n* = 39) der Teilnehmenden, für die eine fortgeschrittene Digitalisierung nicht sehr wichtig war. Eine digitale OP-Planung erfolgt an den Kliniken von 38 % der Teilnehmenden. Eine teilweise digitale OP-Planung ist bei 40 % der Teilnehmenden verfügbar und nur bei 22 % wird die OP-Planung vollständig analog durchgeführt. Dies spiegelte sich auch in den Antworten der offenen Fragen wider. Hier gaben die Teilnehmenden an, digitale Lösungen seien in den Bereichen OP-Planung, Lehre, digitale Patientenakte, Ausbildung, aber auch zur Prozessoptimierung (z. B. Ruf in den Operationssaal, Personalplanung, Stationsplanung) aktuell im Einsatz. Eine Entlastung durch Medizintechnik und Digitalisierung wurde von den Teilnehmenden insbesondere bei administrativen Aufgaben (Arztbriefe schreiben, Dokumentation und Aufklärung) gewünscht. Auch ein digitales Logbuch in der Weiterbildung, die Auswertung von Befunden und die Ergänzung der Weiterbildung um virtuelle Methoden und Simulation wurde für die Zukunft gewünscht.

Bei der Frage nach der Anwendung von generativer KI und ChatGPT gab die Mehrzahl der Teilnehmenden (*n* = 52, 61 %) an, diese noch nie benutzt zu haben. Ärzt:innen in Weiterbildung und Klinikleiter:innen taten dies häufiger als Fach- und Oberärzt:innen. Tab. 2 zeigt die Verteilung der Nutzung generativer KI in Bezug auf die verschiedenen Karrierestufen. Unter denen, die die Systeme bisher benutzt haben, gaben die meisten an, dies für Forschung und Lehre (*n* = 23, 27 %), Administration (*n* = 13, 15 %) und Prüfungsvorbereitung (*n* = 16, 19 %) getan zu haben. In den Freitexten wurden Social Media, statistische Auswertungen und Ausschreibungen als weitere Anwendungsfelder genannt.

**Tab. 2:** Antwortenverteilung zum Einsatz generativer KI (Frage 5)

(%)	Ja	Nein
*Forschung und Lehre*		
Studierende	40	60
Arzt:in in Weiterbildung	43,75	56,25
Facharzt:in	14,29	85,71
Oberarzt:in	14,81	85,19
Klinikleiter:in	35	65
*Administrative Tätigkeiten*		
Studierende	40	60
Arzt:in in Weiterbildung	12,5	87,5
Facharzt:in	0	100
Oberarzt:in	11,11	88,89
Klinikleiter:in	30	70
*Vorbereitung auf Prüfungen/Vorträge*		
Studierende	40	60
Arzt:in in Weiterbildung	18,75	81,25
Facharzt:in	7,14	92,86
Oberarzt:in	11,11	88,89
Klinikleiter:in	25	75
*Gutachten*		
Studierende	0	100
Arzt:in in Weiterbildung	0	100
Facharzt:in	0	100
Oberarzt:in	0	100
Klinikleiter:in	10	90
Noch gar nicht		
Studierende	60	40
Arzt:in in Weiterbildung	43,75	56,25
Facharzt:in	14,29	85,71
Oberarzt:in	77,78	22,22
Klinikleiter:in	40	60

Lediglich 8 (9 %) Chirurg:innen gaben an, dass im Verlauf der Weiterbildung für alle ÄiW eine kurrikuläre Einarbeitung am OP-Roboter stattfindet; 23 (27 %) gaben an, dass es diese zwar gibt, aber nur für ausgewählte ÄiW. In fast ebenso vielen Fällen gibt es keine Einarbeitung im Rahmen eines Kurrikulums (*n* = 22, 26 %). In 31 (37 %) Fällen war kein OP-Roboter in der Abteilung vorhanden.

Ein Standortwechsel zu einem Standort mit mehr Medizintechnik kommt jedoch nur für 16 (19 %) Teilnehmende in Betracht. Die Mehrzahl (*n* = 36, 42 %) lehnt einen Klinikwechsel zugunsten einer besseren Verfügbarkeit von Medizintechnik vollständig ab, 20 (24 %) lehnten diesen eher ab. Einen Vergleich der Karrierestufen sowie die Verteilung an universitären und nichtuniversitären Standorten zeigen Tab. 3 und 4.

**Tab. 3:** Antwortenverteilung der Fragen 4, 6, 7, 8, 11 und 12 zwischen ÄiW und nicht in Weiterbildung befindlichen Teilnehmenden

***(%)***	***1***	***2***	***3***	***4***	***5***	***Mittelwert***	***p‑Wert***
**Bei der Wahl meiner aktuellen Tätigkeitsstätte habe ich viel Wert auf eine fortgeschrittene Digitalisierung (z.** **B. digitale Patientenakte, OP-Roboter) meiner Abteilung gelegt**							
Arzt:in in Weiterbildung	6,25	18,75	12,5	43,75	18,75	3,5	**–**
FA/OA/Leitung	22,95	21,31	9,8	24,59	21,31	3	**–**
Gesamt	*21,18*	*22,35*	*10,59*	*27,96*	*18,82*	*3*	*0,2239*
**Die Planung, Durchführung und Evaluation operativer Eingriffe erfolgt in meiner Abteilung digital**							
Arzt:in in Weiterbildung	25	25	31,25	18,75	0	2,4	*–*
FA/OA/Leitung	13,11	22,95	39,34	19,67	4,92	2,8	*–*
Gesamt	*15,29*	*22,35*	*40*	*17,65*	*4,71*	*2,74*	*0,2266*
**Ich habe bereits darüber nachgedacht, meine Tätigkeitsstätte zu wechseln, an einen Standort an dem mehr Medizintechnik zum Einsatz kommt**							
Arzt:in in Weiterbildung	25	6,25	6,25	43,75	18,75	3,25	*–*
FA/OA/Leitung	8,2	6,56	14,75	21,31	49,18	3,97	–
Gesamt	*11,76*	*7,06*	*14,12*	*23,53*	*42,35*	*3,79*	*0,0608*
***(%)***	***„Ja für alle“***	***„Ja aber nicht für alle“***	***„Nein“***	***„Kein Roboter“***	**–**	**–**	***p‑Wert***
**Im Verlauf der Weiterbildung erfolgt eine kurrikuläre Einarbeitung an OP-Robotern**							
Arzt:in in Weiterbildung	0	37,5	25	37,5	–	–	–
FA/OA/Leitung	13,11	22,95	24,59	39,34	–	–	–
Gesamt	*9,41*	*27,06*	*25,89*	*36,47*	*–*	*–*	*0,7377*
**(%)**	**„Ja“**	**„Nein“**	**„Unentschlossen“**	**–**	**–**	**–**	***p-*Wert**
**Würden Sie sich selbst durch KI-basierte Robotik operieren lassen (analog zum selbstfahrenden Auto)?**							
Arzt:in in Weiterbildung	37,5	25	37,5	–	–	–	–
FA/OA/Leitung	22,95	62,3	14,75	–	–	–	–
Gesamt	*25,89*	*50,59*	*22,35*	*–*	*–*	*–*	*0,6686*
**Würden Sie selbst mithilfe KI-basierter Robotik operieren wollen?**							
Arzt:in in Weiterbildung	68,75	6,25	25	–	–	–	–
FA/OA/Leitung	39,34	31,15	29,51	–	–	–	–
Gesamt	*45,89*	*24,71*	*28,23*	*–*	*–*	*–*	*0,1564*

*FA* Facharzt:in, *OA* Oberarzt:in

**Tab. 4:** Antwortenverteilung der Fragen 4, 6, 7, 8, 11 und 12 zwischen Teilnehmenden universitärer und nichtuniversitärer Tätigkeitsstätten

***(%)***	***1***	***2***	***3***	***4***	***5***	***Mittelwert***	***p‑Wert***
**Bei der Wahl meiner aktuellen Tätigkeitsstätte habe ich viel Wert auf eine fortgeschrittene Digitalisierung (z.** **B. digitale Patientenakte, OP-Roboter) meiner Abteilung gelegt**							
Universitär	37,04	29,63	3,7	14,81	14,81	2,41	**–**
Nichtuniversitär	12,24	20,41	12,24	34,69	24,41	3,31	**–**
Gesamt	*21,18*	*22,35*	*10,59*	*27,96*	*18,82*	*3*	*0,0091*
**Die Planung, Durchführung und Evaluation operativer Eingriffe erfolgt in meiner Abteilung digital**							
Universitär	18,52	40,74	29,63	11,11	0	2,33	*–*
Nichtuniversitär	16,33	14,29	42,86	20,41	6,12	2,86	*–*
Gesamt	*15,29*	*22,35*	*40*	*17,65*	*4,71*	*2,74*	*0,0561*
**Ich habe bereits darüber nachgedacht, meine Tätigkeitsstätte zu wechseln, an einen Standort an dem mehr Medizintechnik zum Einsatz kommt**							
Universitär	25,93	11,11	18,52	14,81	25,93	3,04	*–*
Nichtuniversitär	6,12	4,08	10,20	32,65	46,94	4,10	–
Gesamt	*11,76*	*7,06*	*14,12*	*23,53*	*42,35*	*3,79*	*0,0013*
**(%)**	**„Ja für alle“**	**„Ja aber nicht für alle“**	**„Nein“**	**„Kein Roboter“**	**–**	**–**	***p-*Wert**
**Im Verlauf der Weiterbildung erfolgt eine kurrikuläre Einarbeitung an OP-Robotern**							
Universitär	18,52	33,33	25,93	18,52	–	–	–
Nichtuniversitär	6,122	26,53	24,49	42,86	–	–	–
Gesamt	*9,41*	*27,06*	*25,89*	*36,47*	*–*	*–*	*0,0191*
**(%)**	**„Ja“**	**„Nein“**	**„Unentschlossen“**	**–**	**–**	**–**	***p-*Wert**
**Würden Sie sich selbst durch KI-basierte Robotik operieren lassen (analog zum selbstfahrenden Auto)?**							
Universitär	33,33	33,33	29,63	–	–	–	–
Nichtuniversitär	24,49	57,14	18,37	–	–	–	–
Gesamt	*25,89*	*50,59*	*22,35*	*–*	*–*	*–*	*0,8966*
**Würden Sie selbst mithilfe KI-basierter Robotik operieren wollen?**							
Universitär	66,67	7,41	22,22	–	–	–	–
Nichtuniversitär	38,78	28,57	32,65	–	–	–	–
Gesamt	*45,89*	*24,71*	*28,23*	*–*	*–*	*–*	*0,0573*

Während 22 (26 %) Teilnehmende sich selbst durch KI-basierte Robotik operieren lassen würden, wollen 39 (46 %) selbst Anwender dieser Methode sein; 19 (22 %) Teilnehmende zeigten sich unentschlossen auf die Frage, ob sie sich selbst durch einen KI-gestützten Roboter operieren lassen würden, während 24 (28 %) sich unentschlossen in der Anwendung dieser Technik zeigten.

## Diskussion

Die Ergebnisse dieser Studie geben einen Einblick, wie die Bedeutung der Digitalisierung, die verschiedenen Angebote und Anwendungen digitaler Technologien sowie deren Stellenwert für den Standort in Bezug auf die verschiedenen Karrierestufen bewertet werden. In der Gesamtbetrachtung wurde der Grad der Digitalisierung von den Teilnehmenden als unterschiedlich wichtig bewertet. Auffallend ist dabei, dass die im Berufsleben weiter fortgeschrittenen Teilnehmenden mehr Wert auf einen hohen Grad an Digitalisierung legen. Ein Wechsel der Arbeitsstelle zu einer Arbeitsstelle, an der mehr Medizintechnik zum Einsatz kommt, kommt nur für wenige Teilnehmende infrage. Der Anteil derer, die einen Wechsel in Betracht ziehen würden, ist an Universitätskliniken größer. Dies ist möglicherweise Ausdruck eines besonders hohen Anspruchs an das Innovationspotenzial der Tätigkeitsstätte und des Wunsches, den medizinischen Fortschritt maßgeblich mitzugestalten.

Für den Bereich der Patientenversorgung wurden 2019 bereits legislative Grundlagen für eine zügige Implementation innovativer Tools geschaffen. Mit dem Digitale-Versorgung-Gesetz (DVG) und der Digitale-Gesundheitsanwendungen-Verordnung (DiGAV) wurden digitale Gesundheitsanwendungen (DiGA) definiert und als Regelleistung der gesetzlichen Krankenversicherung (GKV) eingeführt; mit dem Digitale-Versorgung-und-Pflege-Modernisierungs-Gesetz (DVPMG) wurde der neue Leistungsbereich weiter ausgestaltet [9–11]. Für den Bereich der klinischen Aus- und Weiterbildung ist der Stellenwert digitaler Lösungen kaum zu unterschätzen. Wie in dieser Umfrage zu sehen ist, variiert der Grad der Umsetzung jedoch stark. Die Ansatzpunkte reichen von der Schaffung einer einfachen, digitalen und ortsunabhängigen Zugriffsmöglichkeit auf bereits vorhandene Lehrmaterialien über Online- und Hybridveranstaltungen (live und remote), Blended-Learning-Konzepte und der Implementierung von Virtual/Augmented Reality, Social Media und künstlicher Intelligenz [12]. In der Praxis werden diese jedoch oftmals nicht umgesetzt, wie in dieser Umfrage deutlich wurde.

Die Akzeptanz digitaler Tools in der Transformation des Gesundheitssystems ist grundsätzlich hoch [11]. Obwohl bereits häufig über positive und negative Effekte der Digitalisierung selbst sowie Effekte, die den Nutzungsgrad beeinflussen, berichtet wurde, wurden die Erfahrungen der Nutzer:innen, wie z. B. ihre Gedanken und Gefühle, weniger häufig diskutiert [11, 13]. In einer Studie der gewerkschaftsnahen Hans-Böckler-Stiftung von 2017 gaben die Befragten an, dass in der Vergangenheit der Arbeitsdruck als Folge digitaler Technologien tendenziell gestiegen sei [14]. Zwar bewertete eine Mehrheit die Auswirkungen neutral, mehr als ein Drittel sah aber mehr Zeit und Leistungsdruck, während lediglich eine Minderheit zu einer gegenteiligen Einschätzung kam. Die Akzeptanz und die Auswirkungen auf die Berufszufriedenheit sind jedoch auch wichtige Zielvariablen, die im Prozess der Entwicklung und Implementierung mitgedacht werden sollten. Um eine ganzheitliche Sicht auf die digitale Transformation des Gesundheitswesens zu erhalten, besteht auch weiterhin großer Forschungsbedarf, um die Implementierung digitaler Innovationen und den Umgang aller Akteure damit zu evaluieren [4, 14].

Die Mehrheit der Teilnehmenden dieser Umfrage gab an, bisher noch keine KI-basierten Tools verwendet zu haben. KI-Tools können jedoch hilfreich und verantwortungsvoll im wissenschaftlichen Kontext eingesetzt werden, wie z. B. bei sprachlichen Korrekturen und als Formulierungshilfe [15]. Eine Studie von Xie et al. untersuchte verschiedene KI-Plattformen auch im Hinblick auf deren Potenzial, junge Ärzt:innen bei Entscheidungsfindung und Lernen zu unterstützen [4]. Vor allem die wohl bekannteste Plattform, ChatGPT, demonstrierte recht valide Inhalte und Empfehlungen und war in hohem Maße im Einklang mit den Leitlinien. Diese Form der Evaluation ist wichtig, um eine konstruktiv-kritische Auseinandersetzung mit technischen Innovationen zu ermöglichen, deren Integration zu fördern, jedoch auch um auf potenzielle Risiken und Schwächen aufmerksam zu machen. Zu ähnlichen Ergebnissen und Schlüssen kommen auch weitere Studien, die die Anwendung der KI-Plattformen im Rahmen der Patienteninformation und Aufklärung untersuchten [16, 17]. Sinnvolle Anwendung können u. a. die Erstellung von Informationsmaterial, die laienverständliche Darstellung komplexer Sachverhalte und Lifestyleedukation sein. Da jedoch auch substanzielle Risiken durch Falschinformationen und Verständnisprobleme bestehen können, sollte eine professionelle Kontrolle in der Anwendung an und mit den Patienten gegeben sein [16, 18]. Weiterhin bestehen Risiken, die einer breiten und nicht fachlich supervidierten Anwendung entgegenstehen. Eines der Hauptprobleme können die selbstreferenziellen Lernschleifen der Modelle sein, bei denen KI-generierte Inhalte in die Lernalgorithmen einfließen, was die Vielfalt des Datenpools bedroht, möglicherweise Vorurteile verfestigt und die Wirksamkeit der Large Language Models verringert (LLMs) [19].

Robotersimulationstraining bietet große Vorteile in der medizinischen Aus- und Weiterbildung, da Chirurg:innen ihre technischen Fertigkeiten in einer kontrollierten Umgebung verfeinern können, ohne Patient:innen zu gefährden [19]. Ein wesentlicher Vorteil ist zudem die Möglichkeit, u. a. komplexe Eingriffe wiederholt zu üben, was Selbstvertrauen und Präzision steigert und die Patientensicherheit erhöht. Programme wie z. B. das RoboSET des Royal Australasian College of Surgeons kombinieren Roboterplattformen, VR-Integration und synthetische Organmodelle. Dies ermöglicht Chirurg:innen, sich gezielt auf reale Roboteroperationen vorzubereiten [21]. Auch das spielerische Training gewinnt an Bedeutung. So zeigte unter anderem das Urologieprogramm der Columbia University, dass die Integration von Punktesystemen und Teamwettbewerben die Motivation der Chirurg:innen steigerte. Dadurch verbrachten Ärzt:innen mehr Zeit am Simulator, was wiederum ihre Autonomie im Operationssaal steigerte [22]. Fortschritte wie automatisierte Leistungsmessungen bieten realitätsnahe Trainingsbedingungen und ein objektives Feedback, was insbesondere für Weiterbildungsprogramme von großem Wert sein kann. Regelmäßiges Feedback, das von jungen Chirurg:innen häufig gewünscht wird, steigert zudem die Attraktivität von Weiterbildungsstätten [23].

Unsere Umfrage ergab jedoch, dass 37 % der Befragten in ihren chirurgischen Abteilungen keinen OP-Roboter besitzen. Wenn Roboter vorhanden waren, fand nur in 9 % der Fälle eine strukturierte Einarbeitung statt. 27 % der Teilnehmenden berichteten von Kurrikula, die nicht allen Personen in Aus- und Weiterbildung zugänglich sind. Gleichzeitig besteht ein großes Interesse an der Anwendung roboterassistierter Chirurgie im klinischen Alltag. Viele Kliniken in Deutschland können sich OP-Roboter nur leisten, wenn sie über ein entsprechend großes Budget, eine hohe OP-Auslastung und/oder spezielle Fördermöglichkeiten verfügen. Entsprechend setzen sich diese Systeme vor allem in Universitätskliniken und großen Häusern immer mehr durch, da sie die Präzision und Qualität der chirurgischen Versorgung verbessern. Ein häufig angewandtes System zur ökonomischeren Verwendung besteht darin, dass der Roboter von mehreren Disziplinen genutzt wird. Aufgrund der beschriebenen Hürden bei der Beschaffung und der hohen Kosten, die durch eine konsequente Auslastung refinanziert werden müssen, wird der Roboter häufig wie der „heilige Gral“ gehütet und ist daher für jüngere Chirurg:innen nur schwer zugänglich [24–26].

Auch wenn die Umfrage nur einen begrenzten Einblick erlaubt, wird deutlich, dass die digitale Aus- und Weiterbildung in Deutschland in Zukunft stärker fokussiert werden muss. Die Zahl der Weiterbildungsoperationen nimmt ab, minimal-invasive Verfahren und komplexe Patientensituationen nehmen zu. Simulationen, insbesondere mit OP-Robotern, können entscheidend dazu beitragen die chirurgische Ausbildung auf ein neues Niveau zu heben [27].

Chirurg:innen haben klare Erwartungen an ihre Ausbildungsstätten, die über das Erlernen operativer Fertigkeiten hinausgehen. Durch den Generationswechsel haben sich diese Erwartungen verändert, bedingt durch den Fachkräftemangel können die jungen Chirurg:innen diese mit Nachdruck einfordern. Wichtige Faktoren sind strukturierte Aus- und Weiterbildungsprogramme, die sowohl chirurgische Fähigkeiten als auch interdisziplinäre Kompetenzen fördern. Entscheidend ist die Verfügbarkeit erfahrener Mentor:innen, die neben der operativen Ausbildung auch nichtklinische Aspekte wie Kommunikation, Führung und Stressmanagement vermitteln [28–31]. Ein weiterer Wunsch ist mehr Eigenverantwortung und Entscheidungskompetenz. ÄiW profitieren von einer schrittweisen Erhöhung der Autonomie im OP, begleitet von erfahrenen Chirurg:innen. Dies stärkt ihr Selbstvertrauen und bereitet sie auf eigenständige, komplexe Eingriffe vor – ein entscheidender Faktor für den Karrierefortschritt. Durch den richtigen Einsatz von Medizintechnik in der Weiterbildung kann die Eigenverantwortung und Entscheidungskompetenz gesteigert werden und damit zur gewünschten Autonomie im OP führen [32]. Somit könnte die Attraktivität von Ausbildungsstätten für Chirurg:innen erheblich gesteigert werden. Allerdings würden nach Angaben der Befragten nur 19 % einen Standortwechsel allein aufgrund des verstärkten Einsatzes von Medizintechnik in Betracht ziehen. Dies könnte darauf zurückzuführen sein, dass Robotik in Deutschland noch nicht flächendeckend eingesetzt wird und das Wissen über die Vorteile und Anwendungsbereiche noch begrenzt ist.

Medizintechnik und neue digitale Technologien sind ein zentraler Bestandteil des deutschen Gesundheitswesens und werden in nahezu allen Kliniken eingesetzt. Technologien wie Computertomographen, Operationsroboter und KI-gestützte Systeme verbessern Diagnostik, Therapie und Prävention, etwa durch präzisere Bildanalysen oder personalisierte Behandlungsansätze. Sie ermöglichen schnellere Abläufe, minimal-invasive Eingriffe und eine bessere Patientenversorgung durch Echtzeitmonitoring und Telemedizin.

Trotz ihrer Vorteile bergen Medizintechnik und digitale Technologien Herausforderungen: Hohe Anschaffungs- und Betriebskosten belasten Budgets, die ohnehin – besonders in kleinen Kliniken – häufig bereits stark belastet sind [29]. Zudem erfordert die komplexe Technologie Schulungen und kann durch Bedienungsfehler oder Datenschutzrisiken problematisch sein. Regulierungen wie die EU-Medizinprodukteverordnung erhöhen zusätzlich den administrativen Aufwand.

Dabei ist der Einsatz in der ärztlichen Aus- und Weiterbildung in Deutschland auch bei den Umfrageteilnehmenden selten anzutreffen. Dies ist das Ergebnis eines Zusammenspiels von finanziellen Einschränkungen, traditionellen Ausbildungsstrukturen, fehlender Standardisierung und technologischem Widerstand [30].

Eine Umstellung erfordert Investitionen, Veränderungen in der Ausbildungskultur und die Einführung standardisierter Weiterbildungsanforderungen sowie Kontrollen durch die Ärztekammern. Die genannten Veränderungen bedürfen aber vor allem der öffentlichen Förderung. Nur wenn diese gesichert ist, können Medizintechnik und digitale Technologien auch in die Weiterbildungsordnung aufgenommen und dann tatsächlich umgesetzt werden, denn die tatsächliche Umsetzung in der Klinik erfordert Weiterbilder, die diese Techniken beherrschen und die Zeit haben, sich selbst und andere weiterzubilden. Dennoch ist festzuhalten, dass die Weiterentwicklung dieser langfristig durch Früherkennung von Krankheiten, Verkürzung von Heilungszeiten und Innovation in der Forschung zur Effizienz und Kostenreduktion beitragen kann [31].

### Limitationen

Limitationen ergeben sich durch die geringe Teilnehmerzahl, sodass die Ergebnisse der Umfrage nur beschränkt generalisierbar sind. Zudem befanden sich nur knapp ein Viertel der Teilnehmenden zum Analysezeitraum in Weiterbildung. Die hohe Teilnehmerzahl von Seiten der Allgemein- und Viszeralchirurgie lässt sich sowohl durch die besondere Bedeutung der robotischen Chirurgie für dieses Fach erklären als auch durch die dominierende Anzahl an Mitgliedern in der Deutschen Gesellschaft für Chirurgie. Auch wenn es sich nicht um eine repräsentative Stichprobe handelt, präsentiert diese Umfrage erstmals Daten bezüglich des Einflusses und der Bedeutung der Digitalisierung auf die Weiterbildung in der Chirurgie in Deutschland.

## Schlussfolgerung

Die vorliegende Analyse gibt einen Einblick in die Bedeutung und den Einsatz digitaler Technologien in der Chirurgie in Deutschland. Es zeigen sich insbesondere Defizite bei der Anwendung KI-basierter Methoden, einer flächendeckenden Versorgung mit digitalen Technologien sowie dem Zugang von ÄiW zu einer innovativen kurrikulären Weiterbildung. Entgegen der populären Meinung wird ebenso deutlich, dass die Anwendung digitaler Technologien und die begleitende Digitalisierung bislang kein Unterscheidungsmerkmal von Arbeits‑, Aus- und Weiterbildungsstätten ist und bislang zwar das Potenzial eines „Gamechangers“ hat, aber noch nicht flächendeckend implementiert ist. Die Ergebnisse bestätigen auch die Notwendigkeit, das Bewusstsein für die Thematik weiter zu steigern und die Reichweite der DGCH über die sozialen Medien zu erhöhen. Weitere Studien sollten sich insbesondere auf die praktische Umsetzung der Etablierung digitaler Technologien und die begleitende Digitalisierung widmen und hierbei die Bedürfnisse der verschiedenen Berufsgruppen berücksichtigen.

## Fazit für die Praxis

- Es gibt Defizite bei der Anwendung KI-basierter Methoden, einer flächendeckenden Versorgung mit digitalen Technologien sowie dem Zugang von ÄiW zu einer innovativen kurrikulären Weiterbildung.
- Digitale Technologien und die begleitende Digitalisierung sind bislang kein Unterscheidungsmerkmal von Arbeits- und Ausbildungsstätten.
- Es ist dringend notwendig, das Bewusstsein für die Thematik weiter zu steigern.

## Data Availability

Die erhobenen Datensätze können auf begründete Anfrage in anonymisierter Form beim korrespondierenden Autor angefordert werden. Die Daten befinden sich auf einem Datenspeicher der Universität zu Köln.
